# Supplementary material for: Percentile scores for the revised University of Pennsylvania Smell Identification Test for 16,972 individuals 60 years of age and older
Source: NPJ Parkinsons Dis. 2025 Oct 1;11:280. doi: 10.1038/s41531-025-01095-9 (PMC12488959; doi:10.1038/s41531-025-01095-9)

SUPPLEMENTAL MATERIAL

Percentile Scores for the Revised University of Pennsylvania Smell Identification Test for 16,972 Individuals 60 years of Age and Older

Pierz, K.A.; Aamodt, A.; Gochanour, C.; Kurth, R.; Brumm, M.C.; Coffey, C.S.; Heathers, L.E.; Totten, M.; Doty, R.L.; Marek, K.; Siderowf, A.; and the Parkinson's Progression Markers Initiative

Table S1. Demographics and prodromal features of cohort study participants

	**Females by cohort**		**Males by cohort**	
	**PARS/PPMI**[a]	**PPMI Online/ ST Direct**	**PARS/PPMI**[a]	**PPMI Online/ ST Direct**
**N**	4246	11754	3679	5218
**Age**, mean (SD)	67.8 (6.1)	68.0 (5.8)	69.7 (6.7)	69.8 (6.4)
**Race (White)**, n (%)	3974 (97%)	11323 (97%)	3473 (98%)	4969 (97%)
**Ethnicity (Hispanic)**, n (%)	48 (1.5%)	225 (2.0%)	21 (0.7%)	119 (2.4%)
**Family history of PD**, n (%)	1418 (34%)	3286 (28%)	847 (23%)	1310 (25%)
**Used laxatives regularly**, n (%)	424 (10%)	303 (12%)[b]	249 (7%)	189 (10%)[b]
**< 1 bowel movement daily**, n (%)	852 (20%)	561 (23%)[b]	494 (14%)	346 (18%)[b]
**Self-reported hyposmia**, n (%)	537 (13%)	1701 (15%)	574 (16%)	977 (19%)
**RBD features**, n (%)	268 (7%)	1190 (10%)	618 (19%)	1156 (22%)

[a] Original UPSIT scores were derived from a separate cohort than the new cohort reported here, but recruited in a similar manner.[9]
[b] Only available for PPMI Online participants.

Table S2. UPSIT percentile by self-reported race

	Self-Reported Race						
	**American Indian or Alaska Native** (N = 30)	**Asian** (N = 142)	**Black of African American** (N = 114)	**Native Hawaiian Pacific Islander** (N = 7)	**White** (N = 16292)	**Multiracial** (N = 182)	**Missing Race** (N = 205)
**Sex, n (%)**							
Male	8 (27%)	53 (37%)	29 (25%)	1 (NA)	4969 (30%)	65 (36%)	93 (45%)
Female	22 (73%)	89 (63%)	85 (75%)	6 (NA)	11323 (70%)	117 (64%)	112 (55%)
**Age, Mean (SD)**	67.8 (4.5)	67.5 (6.1)	66.7 (4.8)	67.7 (NA)	68.6 (6.1)	67.6 (6.0)	68.0 (5.7)
Median (min, max)	67.5 (60, 78)	66.1 (60, 87)	66.0 (60, 79)	63.1 (NA)	67.6 (60, 95)	65.9 (60, 89)	67.2 (60, 85)
**UPSIT raw score**							
Mean (SD)	33.8 (5.6)	33.7 (5.6)	30.9 (8.1)	31.3 (NA)	33.3 (6.4)	33.0 (6.5)	33.2 (6.0)
Median (min, max)	35 (15, 39)	35 (11, 40)	34 (7, 40)	37 (NA)	36 (2, 40)	35 (13, 40)	35 (7, 40)
**UPSIT Percentile**							
Mean (SD)	48.8 (27.8)	48.9 (26.7)	37.1 (26.3)	49.9 (NA)	50.1 (28.7)	49.7 (31.0)	48.9 (28.3)
Median (min, max)	51.8 (3, 94)	45.0 (1, 100)	31.0 (1, 98)	57.0 (NA)	50.0 (1, 100)	51.8 (1, 99)	45.5 (1, 99)
≤10th Percentile, n (%)	3 (10%)	10 (7%)	23 (20%)	2 (NA)	1669 (10%)	25 (14%)	23 (11%)
≤15th Percentile, n (%)	4 (13%)	17 (12%)	28 (25%)	2 (NA)	2480 (15%)	36 (20%)	32 (16%)

Table S3. UPSIT percentile by self-reported ethnicity

	Self-Reported Ethnicity		
	**Hispanic or Latino** (N = 344)	**Not Hispanic or Latino** (N = 16009)	**Missing Ethnicity** (N = 619)
**Sex, n (%)**			
Male	119 (35%)	4911 (31%)	188 (30%)
Female	225 (65%)	11098 (69%)	431 (70%)
**Age, Mean (SD)**	67.0 (5.3)	68.5 (6.0)	69.5 (6.6)
Median (min, max)	66.1 (60, 87)	67.6 (60, 95)	68.7 (60, 91)
**UPSIT raw score**			
Mean (SD)	33.2 (6.3)	33.3 (6.4)	32.5 (7.1)
Median (min, max)	35 (4, 40)	36 (2, 40)	35 (4, 40)
**UPSIT Percentile**			
Mean (SD)	47.2 (28.1)	50.1 (28.7)	47.9 (29.0)
Median (min, max)	45.0 (1, 100)	50.0 (1, 100)	45.5 (1, 100)
≤10th Percentile, n (%)	37 (11%)	1641 (10%)	77 (12%)
≤15th Percentile, n (%)	61 (18%)	2425 (15%)	113 (18%)

Table S4. UPSIT percentile by self-reported family history

	Family History of PD			
	**No** (N = 11464)	**Yes** (N = 4596)	**Not Sure** (N = 779)	**Missing** (N = 133)
**Sex,** n (%)				
Male	3619 (32%)	1310 (29%)	247 (32%)	42 (32%)
Female	7845 (68%)	3286 (71%)	532 (68%)	91 (68%)
**Age,** Mean (SD)	68.8 (6.2)	67.9 (5.8)	67.7 (5.6)	69.9 (6.1)
Median (min, max)	67.9 (60, 95)	67.0 (60, 94)	66.9 (60, 93)	70.2 (60, 92)
**UPSIT Raw Scores,** Mean (SD)	33.2 (6.5)	33.9 (6.0)	32.1 (7.2)	32.8 (6.6)
Median (min, max)	35 (2, 40)	36 (2, 40)	35 (7, 40)	35 (11, 40)
**UPSIT Percentile**				
Mean (SD)	49.8 (28.8)	51.5 (28.2)	44.4 (29.0)	48.9 (28.5)
Median (min, max)	50.0 (1, 100)	54.0 (1, 100)	42.0 (1, 100)	50.0 (2, 99)
≤ 10th Percentile, n (%)	1199 (10%)	415 (9%)	124 (16%)	17 (13%)
≤ 15th Percentile, n (%)	1779 (16%)	623 (14%)	172 (22%)	25 (19%)

Supplemental Figure 1. Distribution of UPSIT data by version and population (PD and HC). The distribution of UPSIT scores within subjects is depicted for the original version of the UPSIT (red) and the revised version of the UPSIT (blue) for healthy controls (top panel) and for patients with Parkinson's disease (bottom panel).

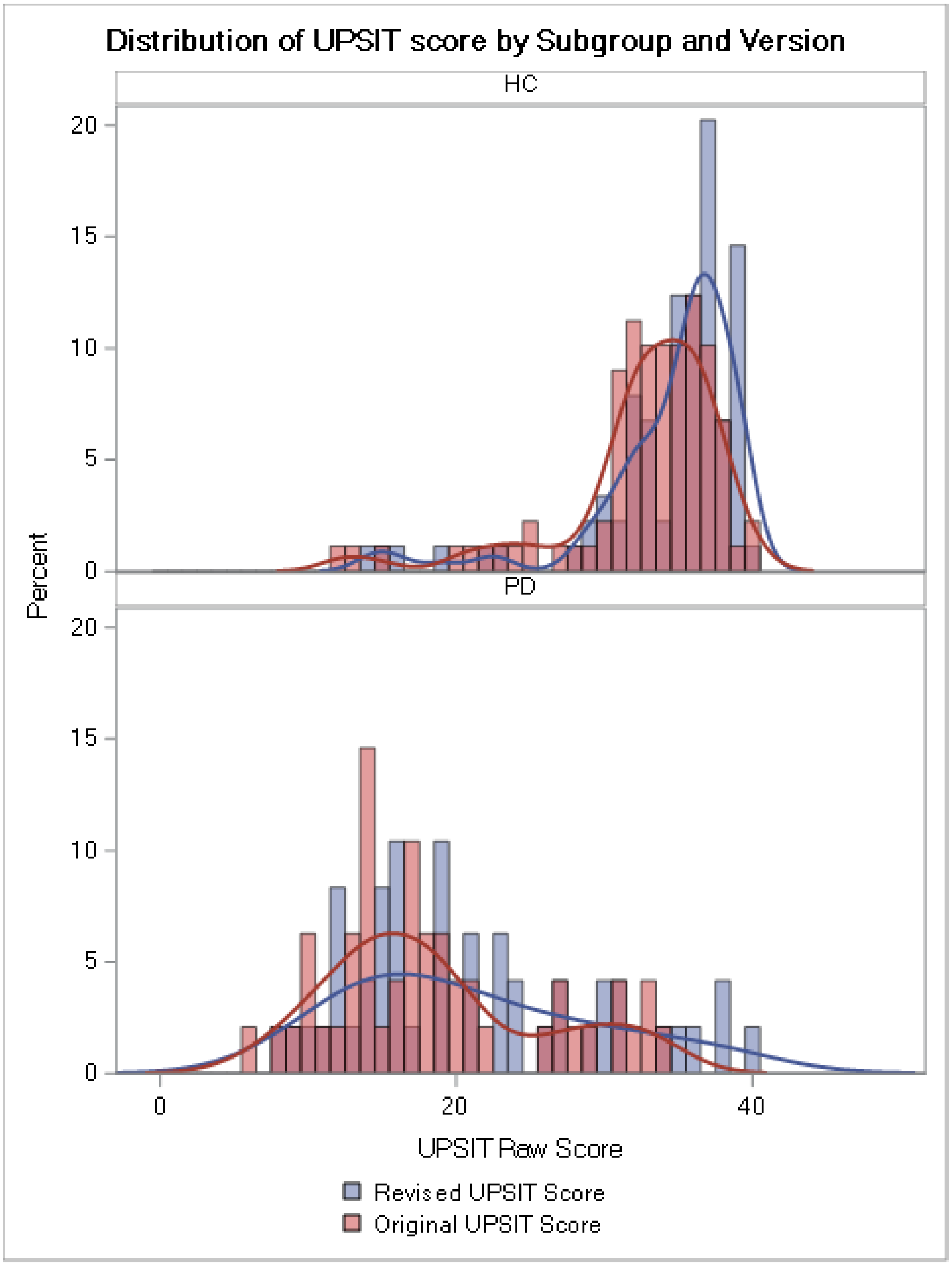

Supplement: Supplementary file 1 — Supplementary Information [file 41531_2025_1095_MOESM1_ESM.pdf]
